# Susceptibility of Human Airway Tissue Models Derived From Different Anatomical Sites to *Bordetella pertussis* and Its Virulence Factor Adenylate Cyclase Toxin

**DOI:** 10.3389/fcimb.2021.797491

**Published:** 2021-12-23

**Authors:** Rinu Sivarajan, David Komla Kessie, Heike Oberwinkler, Niklas Pallmann, Thorsten Walles, Agmal Scherzad, Stephan Hackenberg, Maria Steinke

**Affiliations:** ^1^ Chair of Tissue Engineering and Regenerative Medicine, University Hospital Würzburg, Würzburg, Germany; ^2^ Department of Microbiology, University of Würzburg, Würzburg, Germany; ^3^ Department of Thoracic Surgery, University Medicine Magdeburg, Magdeburg, Germany; ^4^ Department of Oto-Rhino-Laryngology, Plastic, Aesthetic and Reconstructive Head and Neck Surgery, University Hospital Würzburg, Würzburg, Germany; ^5^ Department of Oto-Rhino-Laryngology – Head and Neck Surgery, Rheinisch-Westfälische Technische Hochschule Aachen (RWTH) Aachen University Hospital, Aachen, Germany; ^6^ Translational Center Regenerative Therapies, Fraunhofer Institute for Silicate Research ISC, Würzburg, Germany

**Keywords:** human nasal epithelial cells, human tracheo-bronchial epithelial cells, human airway mucosa tissue models, adenylate cyclase toxin, *Bordetella pertussis*

## Abstract

To study the interaction of human pathogens with their host target structures, human tissue models based on primary cells are considered suitable. Complex tissue models of the human airways have been used as infection models for various viral and bacterial pathogens. The Gram-negative bacterium *Bordetella pertussis* is of relevant clinical interest since whooping cough has developed into a resurgent infectious disease. In the present study, we created three-dimensional tissue models of the human ciliated nasal and tracheo-bronchial mucosa. We compared the innate immune response of these models towards the *B. pertussis* virulence factor adenylate cyclase toxin (CyaA) and its enzymatically inactive but fully pore-forming toxoid CyaA-AC^-^. Applying molecular biological, histological, and microbiological assays, we found that 1 µg/ml CyaA elevated the intracellular cAMP level but did not disturb the epithelial barrier integrity of nasal and tracheo-bronchial airway mucosa tissue models. Interestingly, CyaA significantly increased interleukin 6, interleukin 8, and human beta defensin 2 secretion in nasal tissue models, whereas tracheo-bronchial tissue models were not significantly affected compared to the controls. Subsequently, we investigated the interaction of *B. pertussis* with both differentiated primary nasal and tracheo-bronchial tissue models and demonstrated bacterial adherence and invasion without observing host cell type-specific significant differences. Even though the nasal and the tracheo-bronchial mucosa appear similar from a histological perspective, they are differentially susceptible to *B. pertussis* CyaA *in vitro*. Our finding that nasal tissue models showed an increased innate immune response towards the *B. pertussis* virulence factor CyaA compared to tracheo-bronchial tissue models may reflect the key role of the nasal airway mucosa as the first line of defense against airborne pathogens.

## Introduction

The human nasal cavity, the trachea and the bronchi are parts of the conducting airways and are lined by a pseudostratified, ciliated respiratory epithelium. The main cell types of the respiratory epithelium are progenitor-like basal cells, mucus-producing goblet cells and ciliated cells. This specialized epithelial layer is an important barrier, e.g. against airborne bacteria. *Bordetella pertussis* is a Gram-negative bacterium that elicits whooping cough, a highly contagious respiratory disease in humans, which can be prevented by vaccination. However, in recent years, the number of *B. pertussis* cases has been increasing, for example due to adaptation of the bacteria or waning immunity (Esposito et al., 2019). The main host cell target structures of *B. pertussis* are the kinocilia in the human respiratory tract (Weiss and Hewlett, 1986). The bacteria secrete several virulence factors, such as tracheal cytotoxin, pertussis toxin, pertactin or adenylate cyclase toxin (CyaA) (Nieves and Heininger, 2016). It has been reported that the repeat in toxin hemolytic moiety of CyaA can form small cation selective pores on target cell membranes, which increases permeability. The adenylate cyclase domain of CyaA is then delivered to the cytosol where it is activated by cytosolic calmodulin and causes an unregulated conversion of adenosine triphosphate to cyclic adenosine monophosphate (cAMP). This alters the cellular physiology and suppresses the bactericidal function in host cells (Vojtova et al., 2006; Ohnishi et al., 2008; Masin et al., 2015; Angely et al., 2020).

To study the interaction of *B. pertussis* and its virulence factors with airway target cells, animal and human studies have been performed, although mostly cell line- and primary cell-based model systems have been used (Flak and Goldman, 1999; Guevara et al., 2016; Hasan et al., 2018; Naninck et al., 2018; Graaf et al., 2020; Kessie et al., 2020). Since *B. pertussis* is an obligate human pathogen, cell culture models based on human target cells are considered to be most suitable to study host pathogen interaction. Recent publications show *B. pertussis* adherence and impact on the epithelial integrity in human airway culture models (Steinke et al., 2014; Guevara et al., 2016) and several studies were performed to assess the susceptibility of airway tissue models to CyaA, (Bassinet et al., 2004; Hasan et al., 2018; Bianchi et al., 2021). In the present study, we investigated how CyaA and its enzymatically inactive but fully pore-forming toxoid CyaA-AC^-^ modulated the innate immune response in human primary airway mucosa tissue models. Moreover, we characterized host pathogen interaction of *B. pertussis* with these models.

From a histo-morphological point of view, the ciliated nasal and the tracheo-bronchial mucosa appear similar and some studies suggest that human nasal epithelial cells (HNEC) can be used as surrogates for tracheo-bronchial epithelial cells (HTEC) (McDougall et al., 2008; Thavagnanam et al., 2014; Roberts et al., 2018). However, also some differences were reported *in vitro*: for example, HNEC and HTEC show varying basal interleukin (IL)-6 secretion and differential response after stimulation with lipopolysaccharide (Comer et al., 2012). Since *B. pertussis* targets both the upper and the lower ciliated airways, we further investigated if tissue models based on HNEC and HTEC were susceptible differently to the bacteria and their virulence factor CyaA.

## Materials and Methods

### Donor Details

Specimens from human nasal mucosa were obtained from four male and one female donors, who underwent functional sinus surgery (18-59 years old), to isolate epithelial cells and fibroblasts. Moreover, nasal epithelial cells were isolated from brush biopsies from two female donors (27 and 38 years old) at the Department of Otorhinolaryngology, Plastic, Aesthetic and Reconstructive Head and Neck Surgery of the University Hospital Würzburg. Human tracheo-bronchial tissue samples for epithelial cell and fibroblast isolation were obtained from one male and four female donors (31-77 years old) undergoing elective pulmonary resection at the University Hospital Magdeburg. Written informed consent was obtained beforehand, and the studies were approved by the institutional ethics committees on human research of the Julius-Maximilians-University Würzburg (votes 182/10 and 116/17) and Otto-von-Guericke University Magdeburg (vote 163/17), respectively. Additionally, we used HTEC obtained from three donors (two females and one male, 59-71 years old) and HNEC obtained from three donors (two male and one female, 41-63 years old), which were purchased from Epithelix Sarl (Geneva, Switzerland). **Supplementary Table 1** summarizes anonymized donor information. Both nasal and tracheo-bronchial epithelial cells were used in passage two to build the tissue models.

### Cell Isolation and Cell Culture

All cells were cultured under standard conditions (37°C, 5% CO_2_), and cell culture media were renewed three times per week. Primary human airway epithelial cells (HNEC and HTEC) and fibroblasts were isolated and cultured according to previously published protocols (Schweinlin et al., 2017; Lodes et al., 2020). Airway epithelial cells were grown in Airway Epithelial Cell Growth Medium (AECG, PB-C-MH350-0099, PeloBiotech, Germany). Fibroblasts were grown in DMEM (61965-026, Thermo Fisher Scientific, USA) supplemented with 10% fetal calf serum (P150508, PAN biotech, Germany).

### Human Airway Tissue Models

As a 3D scaffold for tissue model generation, we used decellularized segments of the porcine small intestinal submucosa (SIS), as described elsewhere (Schweinlin et al., 2017; Lodes et al., 2020). Animal research was performed according to the German law and institutional guidelines approved by the Ethics Committee of the District of Unterfranken, Würzburg, Germany (approval number 55.2-2532-2-256). SIS segments were fixed between two cylinders (cell crowns). Each scaffold was seeded with 50,000 nasal or tracheo-bronchial fibroblasts from the apical side and cultured under submerged conditions. The next day, 1.5 x 10^5^ HNEC or HTEC were added from the apical side, and the whole construct was submerged for 48 h. Then, the primary tissue models matured under airlift conditions for 21–31 days until beating kinocilia and mucus production could be observed at the light microscopic level. For cell co-culture, a culture medium mixture made of 50% of AECG and 50% fibroblast culture medium was used. The cell culture medium was changed three times per week. Before each experiment, the apical side was washed with sterile DPBS to remove excess mucus and cell debris, and fresh medium was added to the basal compartment.

### Whole Mount Immunocytochemistry

Differentiated tissue models were washed three times with PBS and fixed with 4% PFA overnight at 4°C. The models were then washed with PBS, blocked and permeabilized with 5% bovine serum albumin (BSA) containing 0.01% TritonX-100 in PBS for 1 h at room temperature. The models were incubated with primary antibodies against ZO-1 (1:1000, 21773-1-AP, ProteinTech, UK) and E-cadherin (1:100, 610181, BD Biosciences, Germany) diluted in 3% BSA in PBS overnight at 4°C to decorate the junctional complexes. For invasion and intracellular location of the pathogens, the models were immunostained with LAMP1 (1:1000; L1418, Sigma-Aldrich, Germany) and pancytokeratin (1:2000; C2562, Sigma-Aldrich, Germany) primary antibodies. The models were counterstained with fluorophore conjugated secondary antibodies after three washes with PBS containing 0.5% Tween-20. The models were then mounted with Flouromount-G with DAPI (00-4959-52, ThermoFisher, Germany) onto glass slides after cutting away excess SIS scaffold. Z-stacks through 25 µm were made with a LEICA SP8 confocal laser scanning microscope (Leica Microsystems, Germany) and processed with LAS-X software (Leica Microsystems, Germany).

### Ciliary Beat Frequency Analysis

The 3D tissue models were unmounted from the cell crowns and fixed onto silicon coated Delta T^®^ Heated culture dishes (10199-956, Bioptechs, USA) with insect pins (EntoSphinx, Czech Republic). The 3D tissue models were then covered with 1 ml prewarmed mixed medium and 15 seconds long videos of beating cilia motion was recorded at 100 Hz frame rate. The videos were recorded with a 40x/0.80 W water immersion Achroplan objective (Nikon GmbH, Japan) and a high-speed video camera (Genie HM640, Teledyne Dalsa, Canada) mounted on a Nikon Eclipse 80i microscope (Nikon GmbH, Japan). The mean ciliary beat frequency was determined from at least 5 different areas of the tissue models using the Fourier transformation of grey level changes overtime with a Matlab (MathWorks, USA) script developed by Prof. Dr. Peter König at the University of Lübeck, Germany (Lindner et al., 2017). The temperature of the medium was maintained at 37°C with the Delta T culture dish controller (Bioptechs, USA) during the recording of the high-speed videos.

### cAMP Assay

The 3D tissue models were incubated from the apical side with 1.0 µg/ml CyaA or CyaA-AC^–^. This concentration of CyaA and its toxoid was previously applied by other groups working with human epithelial cells (Bassinet et al., 2004; Eby et al., 2010; Hasan et al., 2018). Fifty mM Tris pH 8.0, 8 M urea and 2 mM CaCl_2_ (TUC) – treated samples served as controls. After 1 h of incubation, the apical surface was washed with PBS followed by cell lysis with 0.1 M HCl at 95°C for 20 min. The samples were then centrifuged at 3000 rpm for 5 min and cAMP in the supernatant was quantified by a direct competitive ELISA according to the manufacturer’s instructions (ADI-901-066, Enzo life sciences, Germany). The samples were run following the acetylated protocol and five different dilutions were performed for each CyaA treated sample in duplicates to obtain reliable data. The amount of cAMP-HRP bound to the plate was determined by reading the colorimetric HRP activity at OD 405 nm. The measured cAMP values were normalized to the total protein content using the DC™ protein assay (5000113 Bio-Rad, Germany). We performed independent experiments (N=7 for HNEC, N=6 for HTEC), each in duplicates. All the CyaA and CyaA-AC^-^ aliquots that were used for the experiments on IL-6, IL-8 and human beta defensin (HBD)-2 secretion, epithelial barrier properties and histology were also used to run a cAMP assay to make sure that the toxin is active.

### Histological Analyses

Histological work-up and ultrastructural analysis were carried out at the Imaging Core Facility of the Biocenter of the University of Würzburg according to a previously published protocol (Prüfert et al., 2004). In brief, 1 h after incubation with TUC, CyaA or its toxoid, nasal tissue models were washed and fixed in 2.5% glutaraldehyde, then fixed in 2% OsO_4_, washed with H_2_O, and incubated in 0.5% uranyl acetate. Then, the samples were dehydrated and embedded in Epon812. Methylene blue staining was carried out on semi thin sections. Ultrathin sections were imaged at a JEOL JEM-2100 transmission electron microscope equipped with a TVIPS F416 camera or a JEOL JEM-1400 Flash transmission electron microscope equipped with a Matataki Flash camera.

### Fluorescein Isothiocyanate Dextran Permeability Assay

The epithelial barrier integrity of the tissue models was measured by evaluating the flux of fluorescein isothiocyanate (FITC)-conjugated dextran (4 kDa; 46944-100MG-F, Sigma, Germany) from the apical to the basal compartment. Before and after treatment with 1.0 µg/ml CyaA, CyaA-AC^–^ or TUC buffer for 24 h, the apical surface was washed with PBS and 1 ml of fresh co-culture medium was added to the basal compartment. 500 µl of 0.25 mg/ml of FITC-dextran dissolved in 1x DMEM (61965-026, Thermo Fisher Scientific, USA) were incubated on the apical compartment for 30 min protected from light in the incubator. Then, 100 µl of the medium was collected from the basal compartment into a microplate (PS, 96 well, black, F-bottom; 655076, greiner bio one, Germany) in duplicates and analyzed in a TECAN reader (absorbance: 490 nm, emission: 525 nm). The mean absorbance measured in four independent experiments (N=4, each in duplicates) was normalized to a cell-free SIS scaffold (interpreted as 100% permeable or as 0% integrity) mounted on a cell crown. The integrity of the airway mucosa tissue models to FITC-dextran was displayed as percentage values.

### IL-6, IL-8, and HBD-2 Secretion

Three-dimensional human airway mucosa tissue models were washed in PBS to remove debris and excess mucus secretion and then treated from the apical side with 1.0 µg/ml CyaA, CyaA-AC^–^ or TUC buffer for 24 h. The supernatants were collected and analyzed for secreted IL-6 and IL-8 using the human inflammatory cytokine cytometric bead array kit (551811, BD Biosciences, USA) following the manufacturer’s instructions. The supernatants were further analyzed for secreted HBD-2 by ELISA (ARG80903, arigo.biolaboratories, Germany). The assays were performed in independent experiments, each in duplicates (IL-6 and IL-8: N=6 for HNEC, N=5 for HTEC; HBD-2: N=4 for HNEC and HTEC).

### *B. pertussis* Infection Assays

To determine the number of bacteria that adhered to the models after 24 hours infection, the tissue models were developed as previously described and inoculated with *B. pertussis* Tohama 1 wild type strain at an MOI of 50 from the luminal surface. The models were then washed 3x with sterile PBS and incubated with 1% saponin for 20 minutes. Ten-fold serial dilutions of the lysate were made, 100 µl were plated on Bordet-Gengou agar (BGA) and incubated at 37°C. Colonies on the plate were counted and expressed as colony forming units (cfu)/ml. The ability of *B. pertussis* to invade and survive intracellularly in the airway mucosa models was assessed using the polymyxin B protection assay. The infection was allowed to continue for 24 h in a humidified incubator at 37°C. The extracellular bacteria were killed by incubating the models with 100 µg/ml polymyxin B sulfate (P4932, Sigma-Aldrich, Germany) for 2 hours after washing off non-adherent bacteria with sterile PBS. To determine the intracellular survival overtime, the models were washed three times after the treatment with 100 µg/ml polymyxin B to remove the antibiotic and cultured with for a further 5 days mixed media containing 10 µg/ml polymyxin B. The models were lysed by incubating with 1% saponin for 20 minutes and serial dilutions of the lysate were plated on BGA plates to enumerate the cfu. Viable intracellular bacteria were expressed as cfu/ml. For immunofluorescence imaging, the models were infected with a green fluorescent protein (GFP) expressing *B. pertussis* Tohama 1 wildtype strain as described above. The models were then fixed with 4% PFA overnight at 4°C, washed and whole mount staining was performed as previously described. The models were imaged on a Leica SP8 laser scanning confocal microscope. The efficacy of the polymyxin B sulphate was determined to be 100% by incubating 2.0x10^7^ bacteria with 100 µg/ml polymyxin B in mixed medium for 2 hours at 37°C.

### Statistical Analysis

Two-way ANOVA was performed using GraphPad Prism (GraphPad Software, Inc., La Jolla, USA). Dunnett’s multiple comparison test was applied to evaluate the statistical differences between control (TUC) and treatment groups (CyaA and CyaA-AC^–^), differences in the control group (TUC: HNEC vs. HTEC), cell type-specific differences as well as intracellular survival of *B. pertussis* in HNEC and HTEC models. Tukey’s multiple comparisons test was applied to evaluate cell type-specific differences between CyaA treated HNEC and HTEC groups. A Welch’s t-test was also performed to assess the statistical difference between the ciliary beat frequency of HNEC and HTEC models. Unpaired t-test was performed to assess the statistical differences of the adhesion assay data. A p value of ≤0.05 was considered to be significant (*: p≤ 0.05, **: p ≤ 0.01, ***: p≤ 0.001) and a p value of >0.05 but ≤0.1 was considered as a trend (#).

## Results

### Characterization of the Human Airway Mucosa Tissue Models

**Figure 1:**
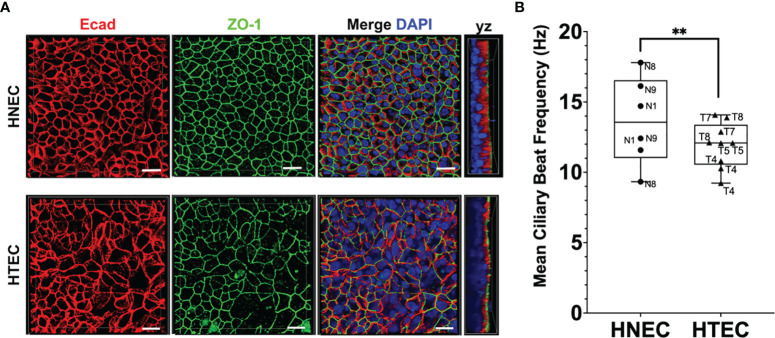
Characterization of ciliated airway tissue models. **(A)** Whole mount immunofluorescent staining of E-cadherin (Ecad) and zonula occludens protein 1 (ZO-1) shows the typical honeycomb organization in both nasal (HNEC) and tracheo-bronchial (HTEC) tissue models cultured at the air-liquid interface for 21 days. Scale bars: 20 µm. **(B)** The mean ciliary beat frequency (CBF) of nasal tissue models was significantly higher compared to tracheo-bronchial models (unpaired t-test, **p ≤ 0.01). Boxplots show means and standard deviations (error bars) of the CBF of at least 30 ciliated cells per tissue model (HNEC-based models: N=3, HTEC-based models: N=4). The graphs also show individual data points to analyze inter- and intra-donor variance.

In the present study, we performed comparative analyses of differentiated nasal and tracheo-bronchial mucosa tissue models using the SIS as a biological scaffold. The tissue models were cultured at the air-liquid interface for 21 to 31 days until beating kinocilia and mucus production was observed at the light microscopic level. To characterize the apical compartment of the tissue models, we first analyzed whole mount double-staining using antibodies against E-cadherin and ZO-1. The samples showed the typical structure of adherens and tight junctions, respectively, and a pseudostratified epithelial layer. Adherens junctions were located directly underneath the tight junctions; HNEC- and HTEC-based tissue models displayed a similar staining pattern (**Figure 1A**). High-speed video analysis of beating kinocilia in viable HNEC- and HTEC-based tissue models revealed a mean ciliary beat frequency of 13.66 ± 3.41 Hz and 12.12 ± 2.73 Hz, respectively. In nasal tissue models, the CBF was significantly higher compared to tracheo-bronchial tissue models (p=0.003, **Figure 1B**). In both HNEC- and HTEC-based tissue models, no noticeable inter-donor variance was observed, however, we found intra-donor variance in nasal models derived from donor N8 (**Figure 1B**). These models are biological replicates and the respective epithelial cells were used in passage 2.

### Susceptibility of Human Airway Mucosa Tissue Models to CyaA and CyaA-AC^-^

**Figure 2:**
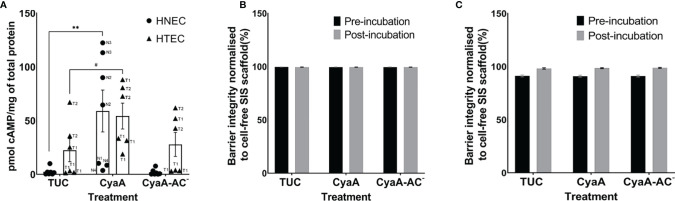
CyaA affects intracellular cAMP level **(A)**, but not the epithelial barrier integrity of nasal (HNEC, **B**) and tracheo-bronchial (HTEC, **C**) airway mucosa tissue models. The samples in **(A)** were incubated with CyaA or its toxoid for 1 h and the samples in **(B)** for 24 h. Data in **(A)** are presented as means ± SEM (N=7 for HNEC, N=6 for HTEC). Each experiment was carried out in duplicates. **p value of Dunnett’s multiple comparison test (TUC vs. CyaA) ≤0.01, #p value of Dunnett’s multiple comparison test (TUC vs. CyaA) >0.05 ≤ 0.1 (trend). The graph also shows individual data points of independent experiments to analyze inter- and intra-donor variance. When no obvious inter- or intra-donor variance was observed, individual data points were not labeled. Data in **(B, C)** are presented as means ± SEM of four independent experiments performed in duplicates (N=4).

**Figure 3:**
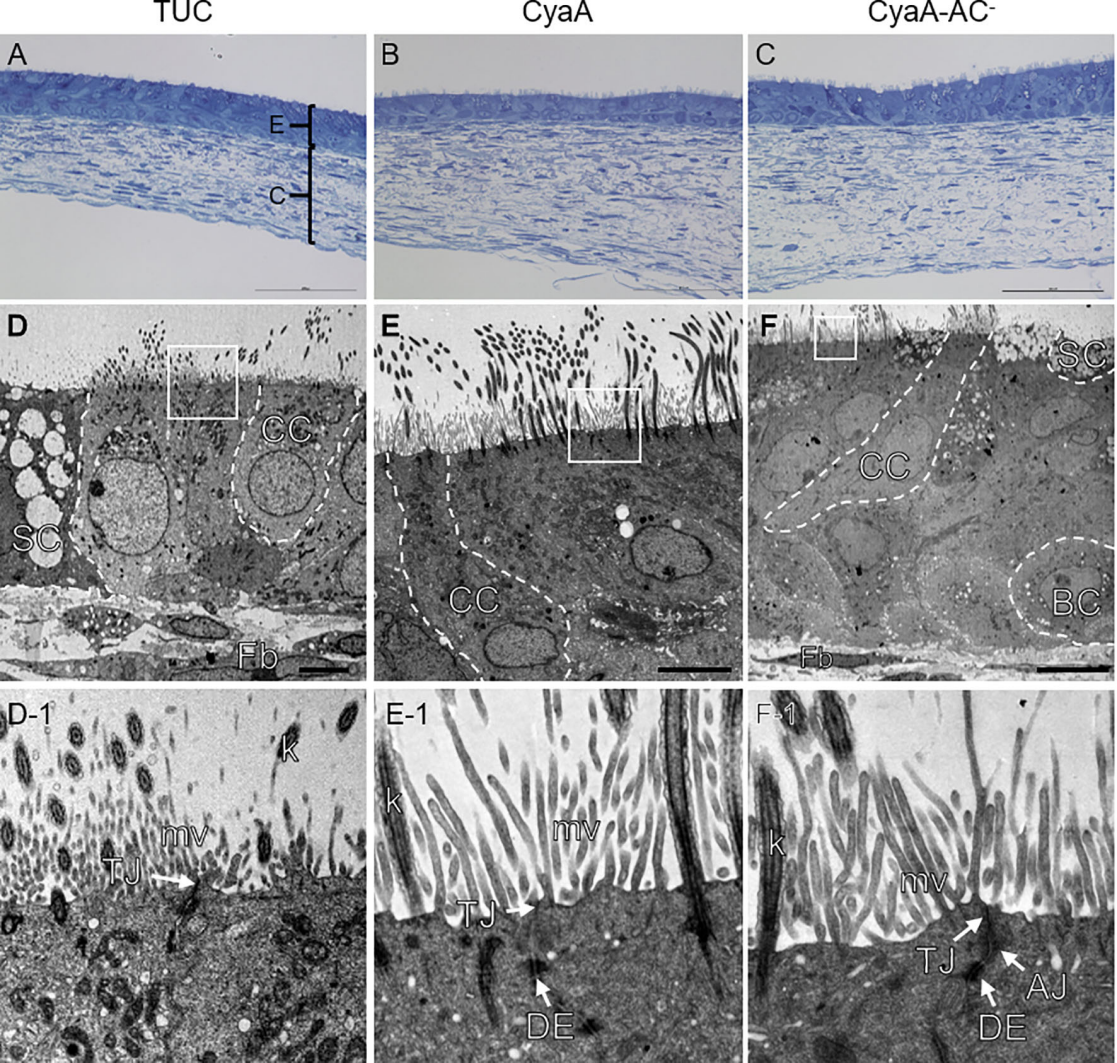
Morphological analysis of nasal mucosa tissue models. Methylene blue staining shows a similar morphology of the tissue models after 1 h TUC **(A)**, CyaA **(B)** and CyaA-AC^-^ treatment **(C)**. Transmission electron microscopic images **(D–F)** display the mucociliary phenotype of the tissue models and inserts (**D-1** – **F-1**) exemplarily confirm intact cell-cell contacts. AJ, adherens junction; BC, basal cell; C, connective tissue; CC, ciliated cell; DE, desmosome; E, epithelial layer; Fb, fibroblast; k, kinocilium; mv, microvilli; SC, secretory cell; TJ, tight junction. Scale bars: **(A–C)**: 100 µm, **(D, E)**: 5 µm, **(F)**: 10 µm.

**Figure 4:**
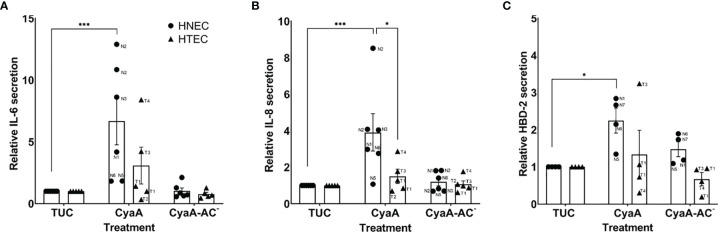
Relative IL-6, IL-8 and HBD-2 secretion of human airway mucosa tissue models after CyaA treatment. CyaA significantly altered relative IL-6 **(A)**, IL-8 **(B)** and HBD-2 secretion **(C)** in nasal tissue models (HNEC), whereas tracheo-bronchial tissue models (HTEC) were not significantly affected. Data are presented as means ± SEM of independent experiments performed in duplicates. **(A, B)** N=6 for HNEC, N=5 for HTEC; **(C)** N=4 for HNEC and HTEC). ***p value of Dunnett’s multiple comparison test (TUC vs. CyaA) ≤0.001, *p value of Dunnett’s multiple comparison test (TUC vs. CyaA) ≤0.05, *p value of Tukey’s multiple comparison test (HNEC vs. HTEC) ≤0.05. The graphs also show individual data points of independent experiments to analyze inter- and intra-donor variance. When no obvious inter- or intra-donor variance was observed, individual data points were not labeled.

After characterization of untreated nasal and tracheo-bronchial mucosa tissue models, we assessed their susceptibility to 1 µg/ml CyaA and CyaA-AC^-^ 1 h (**Figures 2A**, **3**) or 24 h post incubation (**Figures 2B, C**, **4**). To analyze the effects of CyaA and its toxoid on intracellular cAMP, we incubated the tissue models from the apical side and quantified cAMP by ELISA. One hour after incubation, CyaA significantly increased the intracellular cAMP level to 58.9 pmol/mg of total protein (p=0.002) in HNEC-based tissue models and tended to elevate intracellular cAMP level in HTEC-based models (p=0.1) compared to the controls. In CyaA-AC^–^treated samples, no differences compared to controls were observed (**Figure 2A**). Moreover, we found a remarkable inter-donor variation in nasal tissue models: whereas tissue models derived from donors N1 and N4 showed a relatively low response to CyaA treatment, N2 and N3 samples displayed a relatively high intracellular cAMP increase. In HTEC-based tissue models we observed inter-donor variation comparing T1 and T2 and also a high intra-donor variation of T1 (**Figure 2A**).

Since CyaA was able to affect intracellular cAMP level within 1 h, we assessed if the morphology of toxin- and toxoid-treated tissue models changed as well at this time point. To obtain first qualitative insights, we chose nasal tissue models for this experiment. Representative sections of methylene blue-stained nasal mucosa tissue models showed a well-differentiated epithelium that was adjacent to the fibroblast-loaded connective tissue. At the light microscopic level, the overall tissue model morphology appeared intact irrespective of the treatment (TUC, CyaA or CyaA-AC^-^; **Figures 3A–C**). Ultrastructural analysis confirmed the mucociliary phenotype of the tissue models. We identified basal cells, secretory cells, ciliated cells, fibroblasts and verified intact cell cell-contacts, such as tight junctions, adherens junctions and desmosomes (**Figures 3D–F**). Tight junctions appear as electron dense cell-cell contacts located at the very apical compartment of epithelial cells. In some parts of a tight junction, the membranes of two cells are fused to each other and in between there are non-fused spaces. Adherens junctions are located underneath the tight junctions and are characterized by electron dense areas on both sides of the clearly visible intercellular space. Desmosomes are located more basolaterally. Compared to adherens junctions, the intercellular space appears more electron-dense. Comparing the morphology of TUC-, CyaA- and CyaA-AC^–^treated tissue models, no obvious differences were observed 1 h after incubation. We further investigated the epithelial barrier integrity of the models by extending the incubation time with CyaA or the toxoid to 24 h for both nasal and tracheo-bronchial tissue models. Neither incubation with CyaA nor with CyaA-AC^-^ altered the FITC-Dextran transport compared to the controls suggesting that the epithelial barrier was intact (**Figures 2B, C**). In both nasal and tracheo-bronchial tissue models, the normalized barrier integrity values ranged from 89.7% to 99.7% over all experimental conditions. The measured data and normalized percentage barrier integrity values obtained in FITC-Dextran transport assays are shown in **Supplementary Tables 2**–**5**.

CyaA, but not CyaA-AC^-^, had differential effects on cytokine and antimicrobial peptide secretion in human airway mucosa tissue models 24 h after treatment. Whereas HTEC-based tissue models were not significantly affected by CyaA, it significantly increased relative IL-6 (p=0.0008; **Figure 4A**), IL-8 (p=0.0004; **Figure 4B**) and HBD-2 secretion (p=0.02; **Figure 4C**) in HNEC-based tissue models. Moreover, relative IL-8 secretion in nasal tissue models was significantly higher compared to tracheo-bronchial tissue models (p=0.02, **Figure 4B**). Regarding relative IL-6 and IL-8 secretion, we observed inter-donor variance in both nasal and tracheo-bronchial tissue models. The samples based on donors N2, N3, T3 and T4 showed a much higher IL-6 response compared to N1, N5, N6, T1 and T2, respectively (**Figure 4A**). Relative IL-8 secretion was higher in samples based on N1, N2, N3, N6 and T4 compared to N5, T1, T2 and T3, respectively. Moreover, we observed intra-donor variance for N2 (**Figure 4B**). Relative HBD-2 secretion was higher in samples based on donor N1, N6, N7 and T3 compared to N5, T1 and T4, respectively. (**Figure 4C**).

### Infection of Human Airway Mucosa Tissue Models With *B. pertussis*

**Figure 5:**
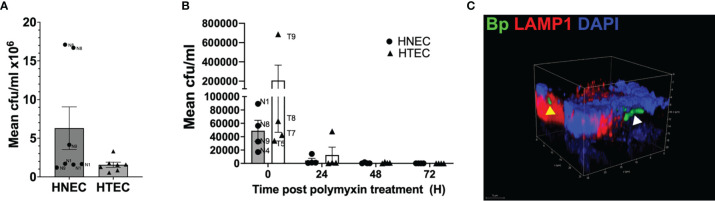
Adherence, invasion and persistence of *B*. *pertussis* in airway mucosa models. Nasal (HNEC) and tracheo-bronchial mucosa tissue models (HTEC) were infected with wild type *B*. *pertussis* Tohama I (MOI: 50) for 24 h. **(A)** Barchart of *B. pertussis-*WT adherence to the HNEC and HTEC model at 24 h post infection. **(B)** The models were treated with polymyxin B (100 µg/ml) for 2 h. The models were washed and incubated with 10 µg/ml polymyxin B for an additional 24, 48, 72 and 96 h. The models were lysed with 1% saponin and the number of viable intracellular bacteria at the indicated time points were assessed by counting the CFU. Data are presented as means + SEM (error bars) of 3 independent experiments performed in duplicates (N=3). **(C)** Confocal image showing the *B. pertussis* (green) colocalized in acidic LAMP1 (yellow arrow head) endosome and nonacidic compartments (white arrow head) in tracheo-bronchial models.

After analyzing the effects of the *B. pertussis* virulence factor CyaA on nasal and tracheo-bronchial mucosa tissue models, we assessed adherence and invasion of *B. pertussis* in its target tissue *in vitro*. The tissue models were inoculated with 10^8^ *B. pertussis* Tohama 1 wild type strain (MOI=50) in 100 µl mixed medium from the apical side. Bacteria adhesion to airway mucosal tissue models was assessed 24 h post inoculation as colony forming units (cfu). The mean cfu/ml (standard error bars) of the cell associated bacteria after the 24 h incubation are shown in (**Figure 5A**). The bacteria seem to preferentially adhere to the HNEC models with 8.16% ± 7.4% of the initial inoculum recovered compared to 1.77% ± 1.1% of the initial inoculum recovered from the HTEC models. In nasal tissue models, we observed inter-donor variance with much higher mean cfu/ml values of N8 compared to N1 and N9. In tracheo-bronchial tissue models no obvious variation was found.

After a 24 h infection period (0 h post polymyxin B treatment), the Tohama I wild type strain was able to invade both HNEC- and HTEC-based tissue models (**Figure 5B**). The persistence of the bacteria intracellularly was also assessed by culturing the models in media containing 10 µg/ml polymyxin B over 5 days. As shown in (**Figure 5B**), after the initial invasion, the cfu of the intracellular bacteria dropped 24 h and even further 48 h after the polymyxin B treatment. No bacteria were recovered from both the HNEC- and HTEC-based tissue models 72 h after polymyxin B treatment. It should however be noted that the intracellular bacteria at 24 h p.i. represents 0.0648% ± 0.049% and 0.367% ± 0.21% of the bacterial cfu inoculum, respectively, in HNEC and HTEC models. A control experiment was setup to determine whether viable *B. pertussis* was shed into the apical compartment by infecting tracheo-bronchial models as previously described and culturing the models for 5 days without polymyxin B after the initial killing of extracellular bacteria with 100 µg/ml polymyxin B. No growth was observed on the BGA plates after 5 days. To determine the localization of the bacteria intracellularly, whole mount staining with the late endosomal/lysosomal marker LAMP1 was performed together with pan-cytokeratin. Our observation from the immunohistochemical staining showed no difference in intracellular survival in the presence or absence of the polymyxin B (**Supplementary Figure 1**). Further analysis of the HTEC models at 24 h post polymyxin B treatment showed the intracellular bacteria localized both in LAMP1-positive vesicles as well as outside them as shown in (**Figure 5C**). Taken together, our data indicate adherence and invasion of *B. pertussis* to both nasal and tracheo-bronchial tissue models. However, due to a high inter-donor variance, especially in tracheo-bronchial tissue models at 0 h post polymyxin B treatment, no significant effects were observed (**Figure 5B**).

## Discussion

In the present study, we assessed the interaction of *B. pertussis* and its virulence factor CyaA with human tissue models of the respiratory mucosa. We compared the results of tissue models representing two different anatomical sites of the airways (nasal *vs*. tracheo-bronchial region). The histological features of nasal and tracheo-bronchial mucosa tissue models were highly similar. *In vitro*, both epithelia consisted of basal, secretory and ciliated cells and displayed a tight barrier, as described previously (Steinke et al., 2014; Schweinlin et al., 2017; Kessie et al., 2020; Lodes et al., 2020). The tissue models showed ciliary beating within the physiological range (Veale et al., 1993) and the kinocilia of nasal mucosa tissue models beat significantly faster than that of the tracheo-bronchial mucosa tissue models. In contrast, others reported no significant differences in ciliary beating when comparing human nasal and bronchial epithelial cell cultures *in vitro* (Devalia et al., 1990; Clary-Meinesz et al., 1992). Even though the differences of measured CBF values were statistically significant in the present study, there is most likely only a minor biological relevance (13.66 Hz *vs*. 12.12 Hz, on average).

An incubation of fully differentiated nasal and tracheo-bronchial mucosa tissue models with 1 µg/ml CyaA revealed a differential susceptibility. Whereas in nasal tissue models, CyaA significantly elevated the intracellular cAMP level, IL-6, IL-8 and HBD-2 secretion, tracheo-bronchial models only tended to be affected or were not affected compared to control samples. Our data on IL-6 secretion in nasal tissue models confirm previous experiments, where CyaA elicited similar effects (Bianchi et al., 2021). To our knowledge, no further publications on the interaction of CyaA with human primary airway epithelial tissue models are available. However, others studied how CyaA affected human airway cell lines: Hasan and co-workers found that CyaA increased neither IL-8 nor HBD-2 secretion in airlifted VA10 cells, a human bronchial epithelial cell line (Hasan et al., 2018), which is in accordance with data obtained from primary HTEC-based tissue models in the present study. In contrast, CyaA increased IL-6 secretion in airlifted VA10 cells (Hasan et al., 2018), which was not observed in our primary tracheo-bronchial tissue models.

The present study further shows that neither 1 h nor 24 h incubation with 1 µg/ml CyaA affected the airway epithelial barrier integrity of the primary cell-based tissue models. In airlifted VA10 cells, CyaA did not disrupt the epithelial barrier after 1 h either. However, 24 h after treatment there was a significant drop in transepithelial electrical resistance (Hasan et al., 2018). A study on the human alveolar A549 cell line showed that CyaA affected for example cell morphology, adhesion and cytoskeleton remodeling, which could also induce changes in airway epithelial barrier integrity (Angely et al., 2020). It appears that human primary airway epithelial cell-based tissue models have a relatively high resistance to CyaA in terms of barrier integrity compared to cell line-based models. However, experimental conditions differed in each study, for example regarding the chosen cell system, scaffold, CyaA concentration and CyaA application (apical *vs*. basolateral). Thus, a direct comparison of the obtained data does not appear feasible. To elucidate possible reasons for such a high resistance to CyaA, further investigations focusing on type III interferons (IFNλ) after CyaA treatment could be conducted. Recent work showed that pre-incubation of human intestinal epithelial cell line-based tissue models with IFNλ could prevent bacteria-induced damage, protect the epithelial barrier integrity, and inhibit bacterial pathogens to cross the epithelial layer (Odendall et al., 2017). Since primary nasal epithelial cells can express high levels of IFNλ following viral infection *in vitro* (Okabayashi et al., 2011), they might also be involved in epithelial protection following incubation with bacterial virulence factors.

This is the first study that shows a differential susceptibility of nasal and tracheo-bronchial human primary airway mucosa tissue models to the *B. pertussis* virulence factor CyaA. Comparing our current results to data obtained from submerged cultures of human primary airway epithelial cells, cell lines or airlifted cell lines, similar but also opposing results were obtained (Bassinet et al., 2004; Hasan et al., 2018; Bianchi et al., 2021). Thus, depending on the chosen airway epithelial cell type (primary cells *vs*. cell lines, nasal *vs*. tracheo-bronchial cells) and cell culture conditions (submerged *vs*. airlifted), certain readouts, such as cytokine secretion, could differ significantly. The actual *in vivo* relevance of a single virulence factor applied to airway tissue models remains to be speculative. During *B. pertussis* infection, several adhesins and toxins act together to attach, colonize and damage host cells effectively. Thus, analyzing the effects of one virulence factor can elucidate only partial characteristics of host-pathogen interaction. Our *in vitro* data show that human nasal tissue is more sensitive to CyaA compared to tracheo-bronchial tissue. Since the nasal mucosa is exposed to airborne pathogens in the first place, a higher innate immune response compared to the lower airways makes sense from a biological perspective. IL-8 is an important chemoattractant recruiting neutrophils to the site of infection. Increased IL-6 secretion seems to play an important role in defending an organism against *B. pertussis* infection: A previous study in mice indicated that IL-6 is involved in leukocyte recruitment and necessary to effectively clear *B. pertussis* from the airways (Zhang et al., 2011). IL-6 is further known to induce mucin5B secretion in human respiratory epithelial tissue models (Chen et al., 2003), which could also support bacterial removal from the respiratory system.

Human nasal tissue cultures have been assessed as surrogates for bronchial models since they can be obtained with minimal invasion by brush biopsies and can be taken from healthy volunteers compared to lower airway epithelial cells. Whereas for example in rhinovirus infection studies similar results for nasal and lower airway epithelial cultures were observed (Alves et al., 2016; Roberts et al., 2018), in airway inflammation studies using culture models of chronic obstructive pulmonary disease patients, nasal epithelial cells could not substitute for bronchial epithelial cells (Comer et al., 2012). In general, it remains to be determined which cell culture system is the most predictive one for the situation *in vivo*, since mostly no direct *in vitro – in vivo* comparisons can be performed. The current opinion is that cell line-based models cultured under 2D conditions do not sufficiently reflect respiratory tract diseases. More complex models, such as 3D human tissue cultures, are assumed to better mimic the physiological conditions *in vivo* in health and disease (Hynes et al., 2020).

*B. pertussis* is considered a classically extracellular pathogen although recent observations suggest that it is capable of cell invasion and persistence. *B. pertussis* was shown to invade and persist intracellularly in HeLa, CHO and human tracheal epithelial cell lines and phagocytic cells (Bassinet et al., 2000; Lamberti et al., 2010; Lamberti et al., 2013; Martín et al., 2015). Our data using primary cell-based tissue models suggest that *B. pertussis* can adhere to and invade both differentiated primary nasal and tracheo-bronchial epithelial cells. Although invasion of the epithelial cells occurs already after 3 hours, Lamberti and co-workers suggested that the process could proceed for at least 24 hours after initial contact of *B. pertussis* with the host cells (Lamberti et al., 2013). Interestingly, although we observed a higher adherence of the bacteria to the nasal mucosa model compared to the tracheo-bronchial models (not statistically significant), the inverse was observed for invasion after 24 hours. The observed difference in the adherence of the bacteria to the HNEC- compared to the HTEC-based tissue models may be due to differences in the distribution of ciliated and non-ciliated cells in these tissues (Crystal et al., 2008; Bustamante-Marin and Ostrowski, 2017). The bacteria preferentially adhere to ciliated cells, which tend to be more abundant in the nasal cavity compared to the tracheo-bronchial tract. The observed difference in the invasion may also be attributed to differences, for example in the cell population, distribution and mucus production in nasal and tracheo-bronchial mucosa. This, however, needs to be studied further to understand the exact mechanism by which the bacteria invade each cell type. Upon invasion, the intracellular bacteria are shuffled into acidic endosomes/lysosomes where they are killed. However, the bacteria can escape lytic killing by sequestering in cholesterol rich nonacidic domains in the host cells (Lamberti et al., 2008; Lamberti et al., 2013; Cafiero et al., 2018). The bacteria, however, do not seem to be able to replicate intracellularly resulting in rapid decline of the bacterial numbers as observed in the present study as reported by Bassinet et al. (2000) using human tracheal epithelial cells and Lamberti and colleagues with A549 cells (Bassinet et al., 2000; Lamberti et al., 2013). *B. pertussis* therefore seems to invade the cells but can only persist intracellular in the airway mucosa tissue models for up to 3 days. The relevance for the overall infection both *in vitro* and *in vivo* therefore remains to be speculative.

Our data obtained from both nasal and tracheo-bronchial tissue models show a certain inter- and intra-donor variance towards both CyaA treatment and *B. pertussis* infection. The individual variance of the respiratory system has been well known and is still a challenge while conducting experimental studies (Veale et al., 1993; Stewart et al., 2012; Ilyushina et al., 2019; Rayner et al., 2019; Bovard et al., 2020). A possible reason for intra-donor variance in independent experiments could be an individual tissue model formation and cell composition. Cell passage is not an issue in the present study, since all the tissue models were built in passage 2. Inter-donor variance can be associated with several factors: since everyone lives in a specific (micro-) environment, the respiratory system is exposed to individual factors, which could impact general cellular physiology, the mucociliary phenotype and/or the innate immune response. For example, nutritional intake, allergies, particle and gas exposure (Koehler et al., 2016), general health condition, but also sex, age and genotype (Saketkhoo et al., 1978; Lee et al., 2012; Pringle et al., 2012; Huang et al., 2016; Uzeloto et al., 2018; Paplinska-Goryca et al., 2021) can all affect the susceptibility of the human airway mucosa and may lead to an individual, biological response both *in vivo* and *in vitro*. Observed inter-donor variance with high- and low-responding samples in our datasets could also represent the situation *in vivo* since there is an individual response for adults towards pertussis infection and the clinical presentation of the disease is influenced for example by age, gender, or time from last pertussis vaccination, as reviewed in (Kilgore et al., 2016).

The present study reveals novel insights into host pathogen interaction of *B. pertussis* and its virulence factor CyaA with primary cell-based human airway mucosa tissue models. Our finding that nasal tissue models showed an increased innate immune response towards CyaA compared to tracheo-bronchial tissue models may reflect the key role of the nasal airway mucosa as first line of defense against airborne pathogens.

## Data Availability Statement

The raw data supporting the conclusions of this article will be made available by the authors, without undue reservation.

## Ethics Statement

Animal research was performed according to the German law and institutional guidelines approved by the Ethics Committee of the District of Unterfranken, Würzburg, Germany (approval number 55.2-2532-2-256). Written informed consent was obtained from the individual(s) for the publication of any potentially identifiable images or data included in this article.

## Author Contributions

RS and DK performed the experiments, discussed the data and co-wrote the manuscript. HO and NP performed the experiments and discussed the data. TW, AS, and SH obtained human tissue specimen for the present study discussed the data and edited the manuscript. MS designed the study, acquired the funding, discussed the data and wrote the manuscript. All authors agree to be accountable for the content of the work. All authors contributed to the article and approved the submitted version.

## Funding

This work was supported by a grant from the Deutsche Forschungsgemeinschaft (DFG GRK 2157; 3D Tissue Models for Studying Microbial Infections by Human Pathogens to MS) and by the Open Access Publication Fund of the University of Würzburg.

## Conflict of Interest

The authors declare that the research was conducted in the absence of any commercial or financial relationships that could be construed as a potential conflict of interest.

## Publisher’s Note

All claims expressed in this article are solely those of the authors and do not necessarily represent those of their affiliated organizations, or those of the publisher, the editors and the reviewers. Any product that may be evaluated in this article, or claim that may be made by its manufacturer, is not guaranteed or endorsed by the publisher.
