# Supplementary material for: Susceptibility of Human Airway Tissue Models Derived From Different Anatomical Sites to Bordetella pertussis and Its Virulence Factor Adenylate Cyclase Toxin
Source: Front Cell Infect Microbiol. 2021 Dec 23;11:797491. doi: 10.3389/fcimb.2021.797491 (PMC8765404; doi:10.3389/fcimb.2021.797491)
Supplement: Supplementary file 1 [file DataSheet_1.docx]

Supplementary Material

## Supplementary Tables

**Supplementary Table 1.** Anonymized donor details. f: female, m: male

|  |  | Nasal specimens | | | | | | | | | Tracheo-bronchial specimens | | | | | | | |
| --- | --- | --- | --- | --- | --- | --- | --- | --- | --- | --- | --- | --- | --- | --- | --- | --- | --- | --- |
| Donor number | 1 | 2 | 3 | 4 | 5 | 6 | 7 | 8 | 9 | 10 | 1 | 2 | 3 | 4 | 5 | 6 | 7 | 8 |
| Gender | m | f | f | m | f | m | m | m | f | m | f | f | m | f | f | m | f | f |
| Age | 26 | 38 | 27 | 18 | 41 | 63 | 50 | 45 | 59 | 49 | 71 | 59 | 62 | 68 | 52 | 65 | 77 | 31 |

**Supplementary Table 2.** Mean absorbance at 490 nm of basal cell culture medium after performing 4 kDa FITC Dextran assays in nasal airway mucosa tissue models. Blank: cell-free small intestinal submucosa

|  | Pre-incubation | | | | | Post-incubation | | | | |
| --- | --- | --- | --- | --- | --- | --- | --- | --- | --- | --- |
| Blank | 39989 | 41612 | 41612 | 41612 | 41612 | 42659 | 41197 | 41197 | 41197 | 41197 |
| TUC | 126 | 232 | 214 | 192 | 155 | 464 | 158 | 177 | 151 | 162 |
| CyaA | 118 | 245 | 359 | 166 | 167 | 479 | 273 | 146 | 167 | 152 |
| CyaA-AC^-^ | 108 | 325 | 174 | 226 | 170 | 459 | 199 | 158 | 159 | 182 |

**Supplementary Table 3.** Mean absorbance at 490 nm of basal cell culture medium after performing 4 kDa FITC Dextran assays in tracheo-bronchial airway mucosa tissue models. Blank: cell-free small intestinal submucosa

|  | Pre-incubation | | | | Post-incubation | | | |
| --- | --- | --- | --- | --- | --- | --- | --- | --- |
| Blank | 43721 | 43721 | 43721 | 43721 | 36354 | 36354 | 36354 | 36354 |
| TUC | 4109 | 4182 | 4439 | 2748 | 1197 | 937 | 269 | 295 |
| CyaA | 4388 | 4262 | 4507 | 2830 | 1089 | 577 | 327 | 276 |
| CyaA-AC^-^ | 4401 | 4110 | 4414 | 2738 | 982 | 277 | 325 | 301 |

**Supplementary Table 4.** The normalized percentage barrier integrity values of nasal airway mucosa tissue models calculated after performing 4 kDa FITC Dextran assays. Blank: cell-free small intestinal submucosa

|  | Pre-incubation (%) | | | | | Post-incubation (%) | | | | |
| --- | --- | --- | --- | --- | --- | --- | --- | --- | --- | --- |
| Blank | 0 | 0 | 0 | 0 | 0 | 0 | 0 | 0 | 0 | 0 |
| TUC | 99.7 | 99.4 | 99.5 | 99.5 | 99.6 | 98.9 | 99.6 | 99.6 | 99.6 | 99.6 |
| CyaA | 99.7 | 99.4 | 99.1 | 99.6 | 99.6 | 98.9 | 99.3 | 99.6 | 99.6 | 99.6 |
| CyaA-AC^-^ | 99.7 | 99.2 | 99.6 | 99.5 | 99.6 | 98.9 | 99.5 | 99.6 | 99.6 | 99.6 |

**Supplementary Table 5.** The normalized percentage barrier integrity values of tracheo-bronchial airway mucosa tissue models calculated after performing 4 kDa FITC Dextran assays. Blank: cell-free small intestinal submucosa

|  | Pre-incubation (%) | | | | Post-incubation (%) | | | |
| --- | --- | --- | --- | --- | --- | --- | --- | --- |
| Blank | 0,0 | 0,0 | 0,0 | 0,0 | 0,0 | 0,0 | 0,0 | 0,0 |
| TUC | 90.6 | 90.4 | 89.8 | 93.7 | 96.7 | 97.4 | 99.3 | 99.2 |
| CyaA | 90.0 | 90.3 | 89.7 | 93.5 | 97.0 | 98.4 | 99.1 | 99.2 |
| CyaA-AC^-^ | 89.9 | 90.6 | 89.9 | 93.7 | 97.3 | 99.2 | 99.1 | 99.2 |

## Supplementary figure caption

**Supplementary figure 1.** Intracellular *B. pertussis* do not proliferate: HTEC-based tissue models were inoculated with GFP_*B. pertussis* at MOI 50 for 24 hours (H). Extracellular bacteria were killed by incubating with 100 μg/ml polymyxin B for 2 H. The tissue models were then washed thoroughly with sterile PBS and incubated for 5 days with and without 10 µg/ml polymyxin B. The HTEC-based tissue models were fixed with 4%PFA at 4°C overnight at 48 H, 72 H, 96 H and 120 H after polymyxin B treatment, immunostained with LAMP1 (red signal) and panCK (white signal). Z-stack of whole model mounts through 25 μm was imaged using TCS SP8 and 3D reconstructed. Viable bacteria were detected up to 72 H after the initial 100 μg/ml polymyxin B in both setups.
