# Supplementary figures and images for: Susceptibility of Human Airway Tissue Models Derived From Different Anatomical Sites to Bordetella pertussis and Its Virulence Factor Adenylate Cyclase Toxin
Source: Front Cell Infect Microbiol. 2021 Dec 23;11:797491. doi: 10.3389/fcimb.2021.797491 (PMC8765404; doi:10.3389/fcimb.2021.797491)

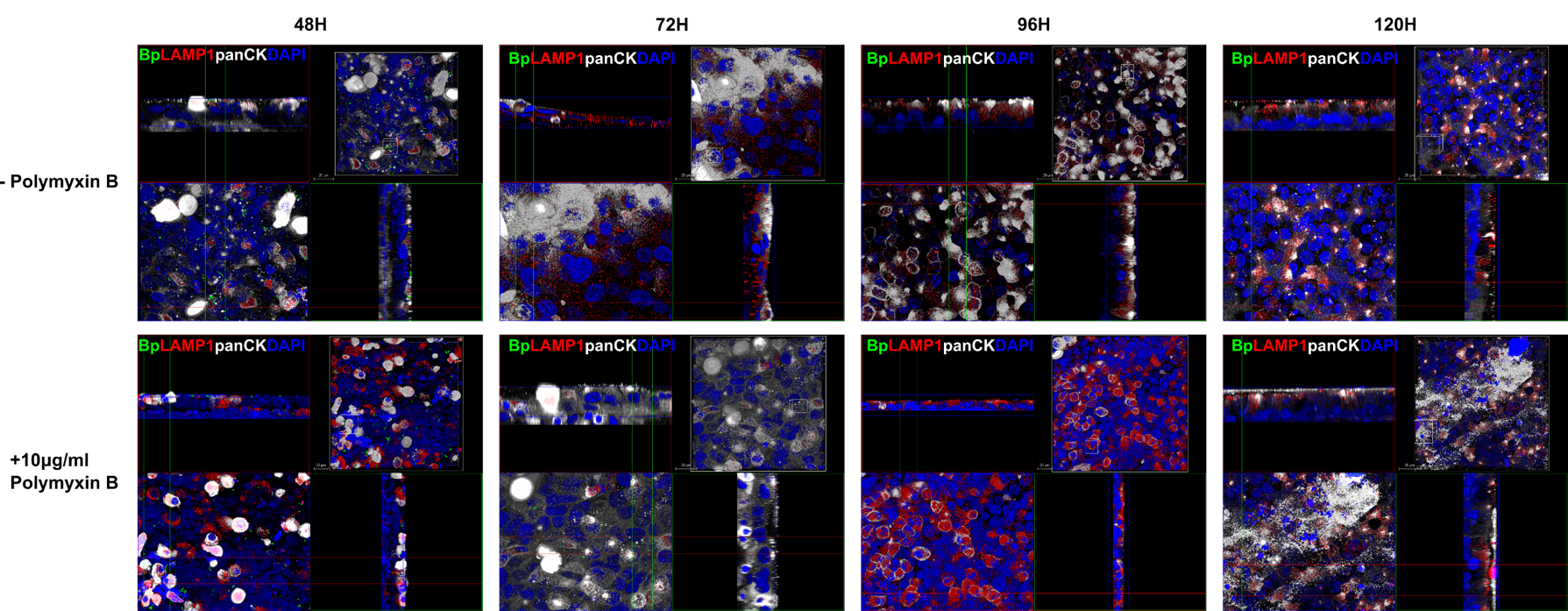

Supplement: Supplementary file 2 [file Image_1.tif]
